# Mental health and the COVID-19 pandemic

**DOI:** 10.1017/ipm.2020.72

**Published:** 2020-09

**Authors:** B. Gavin, J. Lyne, F. McNicholas

**Affiliations:** 1 Department of Child & Adolescent Psychiatry, SMMS, University College Dublin, Dublin, Ireland; 2 Wicklow Mental Health Services, Newcastle Hospital, Greystones, Co. Wicklow, Ireland; 3 Royal College of Surgeons in Ireland, 123 St. Stephen’s Green, Dublin 2, Ireland; 4 Children Health Ireland, Crumlin, Dublin 12, Ireland; 5 Lucena Clinic Rathgar, Dublin 6, Ireland

**Keywords:** COVID-19, mental health, Special Issue, telepsychiatry

## Abstract

With the advent of the COVID-19 pandemic, we have witnessed the greatest global challenge in a generation. The full extent of the mental health impact is, as yet, unknown, but is anticipated to be severe and enduring. In this Special Issue dedicated to mental health and the COVID-19 pandemic, we aim to lay the foundation for an improved understanding of how COVID-19 is affecting mental health services both in Ireland and globally. This Special Issue highlights how the mental health effects of COVID-19 stretch to almost every element of society. The issue includes perspectives from several countries across multiple disciplines and healthcare settings. The drive for rapid innovation and service development is clearly evident throughout and provides hope that by working collaboratively we can positively impact population mental health in the months and years ahead.

## Introduction

The COVID-19 pandemic has brought the greatest global challenge in a generation. The extent and ultimate impact of this pandemic on global health, world economies, societal cohesion and daily life is as yet unknown. The unpredictable nature of the spread of this virus has brought great uncertainty within societies as our knowledge develops about the nature of this virus and its interplay with societal responses (Atchison *et al.* 2020; Verity *et al.* 2020).

This Special Issue of *Irish Journal of Psychological Medicine* provides unique perspectives on mental healthcare in COVID-19, the effects of which stretch to almost every element of society. The papers included in this Special Issue describe the extraordinary challenges of this pandemic across multiple mental healthcare settings, and the admirable efforts to rapidly adapt service delivery to ensure continuity of accessible care for all. Widespread innovation and development are on display across several continents.

## Mental health effects of COVID-19

It is assumed, based on extant data from previous pandemics, together with emerging data from this pandemic, that psychological morbidity will inevitability rise (Maunder *et al.* 2008; The Academy of Medical Sciences, 2020). Furthermore, this morbidity may peak later and endure for longer than the physical health consequences of the pandemic (Gunnell *et al.* 2020). This trend is seen in several perspective pieces in this edition where it is described that the early phases of the pandemic did not necessarily herald an increase in mental health presentations. It is clear, however, that adaptation to the new conditions imposed by COVID-19 has increased workloads on the frontline of mental health. Furthermore, the anticipated increase in mental illness, with potential also for increased suicidality, is deemed most likely in the mid- and post-pandemic phase, as economic contraction, constrained mental healthcare resources, individual vulnerabilities and the stark reality of dramatically altered lifestyles coalesce.

This Special Issue considers interwoven themes across several countries – the psychological impact on those who are carers and those who are cared for; the strain imposed on acute and long-stay settings; the spectrum of vulnerability and resilience to stress amongst the general population, healthcare providers and those with enduring mental health conditions. Efforts to adapt and provide excellent care at this unprecedented time are evident across multiple mental health disciplines.

A major concern now is that the mental health fallout will have a disproportionate impact on society’s most vulnerable and disadvantaged, such as those with pre-existing mental health conditions (Yao *et al.* 2020). While empirical data are scant, it appears reasonable to assert that the widespread impact of a pandemic on mental healthcare systems had not been widely considered by psychiatrists prior to COVID-19. This has resulted in the rapid need to address a myriad of complex unanticipated scenarios, often with high-risk outcomes. This was epitomised by reports of children in residential settings being returned to their families without adequate safety planning or transitional supports in place (Goldman *et al.* 2020). With these issues in mind, there have justifiably been calls for increased resourcing of mental health services, along with a collaborative international effort to address issues such as suicide prevention (Gunnell *et al.* 2020).

## Frontline workers

Frontline workers such as police and healthcare staff are another group who are potentially vulnerable to mental health effects during COVID-19 (Chen *et al.* 2020). Several articles in this issue highlight the critical importance of self-care among these frontline workers in COVID-19. This has also been highlighted in a series of recommendations on COVID-19-related research priorities for mental health (Holmes *et al.* 2020).

Governments worldwide have appealed to the altruism of individuals to work collectively to implement stringent public health measures. Reassuringly, such altruism and sense of purpose, known to act as protective factors to mental ill health, may potentially offset some of the anticipated psychological morbidity (Post, 2005; Kingsbury *et al.* 2019; Brooks *et al.* 2020). However, there is unquestionably an urgent need to develop an understanding of how we can assist frontline workers and their families to utilise adaptive coping strategies both in the short-term and to enhance future pandemic preparedness (Duan & Zhu, 2020; Gavin *et al.* 2020). To develop such interventions in an empirical fashion, it has been recommended to investigate the causal and modifiable factors which can influence pandemic resilience. Such factors may include exercise, prosocial behaviours, conflict resolution and peer group-based interventions. When clear strategies have been identified, it will be crucial to adequately resource frontline staff supports to ensure continued work ethic and morale is maintained. Hopefully, this will come to pass if the pandemic provokes a paradigm shift in thinking as to the centrality of mental health in our global response to this crisis.

## Distribution of finite resources

It is particularly important in this action-oriented phase of pandemic response that due care is given to ensuring that limited mental healthcare resources are utilised in an evidenced-based manner. There are dangers associated with rushing to implement novel psychological support innovations for pandemic-related stress if they are not robustly evaluated and economically viable in the longer term (The Lancet Psychiatry, 2020). In essence, this could potentially involve inappropriate redirection of our scant mental health resources. Against this backdrop, the pandemic should not be seen as merely a crisis to be endured, but also as a unique opportunity to progress targeted research and application of evidenced-based innovation. These sentiments are proposed in the collection of papers in this issue, with many examples and evaluations of service innovation as well as the progression of novel technologies such as providing remote consultation.

## Future research

As the COVID-19 pandemic progresses, it is imperative that priority is given to evaluation of how mental health effects will evolve, and furthermore how to best address them. In particular, we need to determine how we can mitigate the mental health consequences for vulnerable groups. One proposal in this regard has been to use a multidimensional or syndemics approach which addresses the complexities of multiple interacting mental health determinants (Holmes *et al.* 2020). During service reform, it is also crucial to involve those with lived experience. Using an interdisciplinary approach within a coordinated national framework, we can ensure effective, efficient translational innovation.

The urgent need to learn more about the impact of public health measures on general population psychological wellbeing is further emphasised by ethical considerations proposed in relation to population ‘lockdowns’ (Upshur, 2003). The principle of reciprocity holds that if a society demands that individuals withdraw their liberties for the common good, then individuals must be provided with adequate psychological support to discharge that obligation. It is thus essential that policymakers strongly support research to help us understand the mental health reforms needed to deliver effective mental healthcare provision in this dramatically altered landscape.

## Conclusion

We sincerely hope that this Special Issue dedicated to mental health in COVID-19 provides an initial step towards understanding the mental health needs at this extraordinary time both in Ireland and on the international stage. We are very grateful to all contributors who spent time reflecting and submitting to this journal. There is an overwhelming sense that the contributors want to work together to overcome the challenges we face. While it is heartening to witness the resilience demonstrated by those working at the frontline in mental health, it is now essential that there is adequate postpandemic resourcing of mental health to provide care effectively. This Special Issue highlights that through adversity we can achieve change and that by working together we can improve population mental health in the months and years ahead.

## References

[ref1] Atchison CJ , Bowman L , Vrinten C , Redd R , Pristera P , Eaton JW , Ward H (2020). Perceptions and behavioural responses of the general public during the COVID-19 pandemic: A cross-sectional survey of UK adults. medRxiv. doi:10.1101/2020.04.01.20050039 PMC7783373 33397669

[ref2] Brooks SK , Webster RK , Smith LE , Woodland L , Wessely S , Greenberg N , Rubin GJ (2020). The psychological impact of quarantine and how to reduce it: rapid review of the evidence. Lancet 395, 912–920. doi:10.1016/S0140-6736(20)30460-8 32112714 PMC7158942

[ref3] Chen Q , Liang M , Li Y , Guo J , Fei D , Wang L , He L , Sheng C , Cai Y , Li X , Wang J , Zhang Z (2020). Mental health care for medical staff in China during the COVID-19 outbreak. The Lancet Psychiatry 7, e15–e16. doi:10.1016/S2215-0366(20)30078-X 32085839 PMC7129426

[ref4] Duan L , Zhu G (2020). Psychological interventions for people affected by the COVID-19 epidemic. Lancet Psychiatry 7, 300–2. 49. 32085840 10.1016/S2215-0366(20)30073-0 PMC7128328

[ref5] Gavin B , Hayden J , Adamis D , McNicholas F (2020). Caring for the Psychological Well-Being of Healthcare Professionals in the COVID-19 Pandemic Crisis, Ir Med J 113, 51 32268045

[ref6] Goldman PS , Van Ljzendoorn MH , Sonuga-Barke EJS (2020). Lancet Institutional Care Reform Commission Group. The implications of COVID19 for the care of children living in residential institutions. Lancet Child Adolesc Health. doi:10.1016/S2352-4642(20)30130-9 PMC7173818 32330432

[ref7] Gunnell D , Appleby L , Arensman E , Hawton K , John A , Kapur N , Khan M , O’Connor RC , Pirkis J , the COVID-19 Suicide Prevention Research Collaboration (2020). Suicide Risk and prevention during the COVID19 pandemic. Lancet Psychiatry. doi:10.1016/S2215-0366(20)30171-1

[ref8] Holmes EA , O’Connor RC , Perry VH , Tracey I , Wessely S , Arseneault L , et al. (2020). Multidisciplinary research priorities for the COVID-19 pandemic: a call for action for mental health science. The Lancet Psychiatry 7, 547–560. doi:10.1016/S2215-0366(20)30168-1 32304649 PMC7159850

[ref9] Kingsbury M , Clayborne Z , Colman, I , Kirkbride, J (2019). The protective effect of neighbourhood social cohesion on adolescent mental health following stressful life events. Psychological Medicine 1–8. doi:10.1017/S0033291719001235 PMC7322549 31179962

[ref10] Maunder RG , Leszcz M , Savage D , Adam MA , Peladeau N , Romano D , Rose M , Schulman B (2008). Applying the lessons of SARS to pandemic influenza: an evidence-based approach to mitigating the stress experienced by healthcare workers. Canadian Journal of Public Health 99, 486–488. doi:10.1007/BF03403782 19149392 PMC5148615

[ref11] Post SG (2005). Altruism, happiness, and health: it’s good to be good. International Journal of Behavioral Medicine 12, 66–77. 15901215 10.1207/s15327558ijbm1202_4

[ref12] The Academy of Medical Sciences http://www.acmedsci.ac.uk/COVIDmentalhealthsurveys Date accessed: May 2020.

[ref13] The Lancet Psychiatry (2020). Send in the therapists. Lancet Psychiatry 7, 291. doi:10.1016/S2215-0366(20)30102-4 32199496 PMC7103927

[ref14] Verity R , Okell LC , Dorigatti I , Winskill P , Whittaker C , Imai N , et al. (2020). Estimates of the severity of coronavirus disease 2019: a model-based analysis. The Lancet Infectious Diseases 20, 669–677. doi:10.1016/S1473-3099(20)30243-7 32240634 PMC7158570

[ref15] Upshur R (2003). The Ethics of Quarantine. Medicine and Society. (5) 11. 10.1001/virtualmentor.2003.5.11.msoc1-0311 23267516

[ref16] Yao H , Chen JH , Xu YF (2020). Patients with mental health disorders in the COVID 19 epidemic. Lancet Psychiatry 7, e21. 32199510 10.1016/S2215-0366(20)30090-0 PMC7269717

